# Exploring the Influencing Factors on Living Alone and Social Isolation among Older Adults in Rural Areas of Thailand

**DOI:** 10.3390/ijerph192114572

**Published:** 2022-11-06

**Authors:** Pawinee Iamtrakul, Sararad Chayphong

**Affiliations:** Center of Excellence in Urban Mobility Research and Innovation, Faculty of Architecture and Planning, Thammasat University, Paholyothin Street, Pathumthani 12120, Thailand

**Keywords:** activities of daily living, aging society, chronic diseases, socioeconomic status, urbanization

## Abstract

Older adults living alone present a vulnerable physical and mental health group with public health and service needs. This situation has risen and is therefore expected to increase calls for urgent attention from concerned authorities. This article focuses on the study of factors related to different living arrangements of older adults and also examines the extent to which baseline variables explained the association between living alone and social isolation characteristics. A questionnaire survey restricted to respondents aged 60 years and over, living in Ban Phaeo, Samutsakhon, Thailand, was scoped for data collection. Older adults living alone and in co-residence (living together) constitute a total of 1162 samples. The binary logistic regression model was applied to examine the association between living alone and social isolation characteristics. The result found that factors relating to older adults’ different living arrangements are marital status, household members numbers, level of dependency, and type of caregivers. An association was found between the characteristics of living alone and social isolation in three relative variables, which are age, activities of daily living (ADLs), and type of caregivers. In conclusion, household living arrangements have different related factors like marital status, where a single or divorced person is more likely to live alone. Furthermore, it is also influenced by the need for caregiving on the part of the older adult or family members; particularly, their children typically emerge as the unpaid assistance from families. When only a sample of older adults living alone with social isolation is considered, it was discovered that with the advancing age of older adults living alone, whether single or married, encountered problems with the activities of daily living (ADLs). This set of people rarely goes out to perform activities outside their home and seldom attend social and physical activities. This could lead to a risk of social isolation with a greater risk of physical and mental health problems, including the well-being of older adults living alone in later life. Thus, family caregivers play a key role as a primary source of support to prevent older adults from being socially isolated, which has become an integral part of our healthcare system in promoting physical, mental, and functional health among older adults in a positive way.

## 1. Introduction

Many countries worldwide are facing the problem of an aging society, showing that the global population is aging rapidly [1]. Between 2015 and 2050, the proportion of the world’s population above 60 years will rise to nearly double, from 12% to 22% [2]. The projections indicate that in 2050 there will be a more significant number of older people aged 60 or over [3]. The aging of the global population is the most critical medical and social demographic problem worldwide. Many countries are looking for ways to deal with an aging society, prompting the UN declaration on the Decade of Healthy Aging, 2021–2030, as a main strategy to achieving and supporting actions to build a society for all ages [4]. Asia has been facing a massive demographic shift worldwide by 2050; the number of people in Asia aged 65 or older is expected to grow to 937 million—more than double today’s number [5]. Thailand has been an aging society since 2005 and is expected to be a super-aged society by about 2032 [6]. The projection of the population aged 60 and above increased from 13% in 2010 to 33% in 2040. Thailand is one of the fastest aging countries in the world, while rapid urbanization has influenced on the health and quality of older adults’ life [5]. This posed significant challenges in terms of meeting the needs of an aging society: a shifting disease burden, increasing expenditure on health and long-term care, labor force shortages, dissaving, living alone, social isolation, and potential problems with old-age income security [7,8]. Because everyone requires some care and support, increasing life expectancy and declining birth rates are considered significant achievements in modern science and health care with significant impacts in current and future generations.

Living alone and in isolation are significant challenges and problems in an aging society, capable of impacting older adults significantly, both physically and emotionally. It has a strong correlation to older people’s well-being, which is as important in older age as at any other time of life [9,10]. Older people who live alone are more likely to be less socially engaged and have decreased physical activity, and living alone in old age is becoming increasingly common [11,12,13]. Longer life spans, the availability of pensions or retirement income, population mobility, divorce rates, no children, a preference to live independently as long as their health is good enough, and being unmarried all justify this trend [14,15,16,17]. Living alone can present many challenges, such as being poor when older, and the poverty is more likely to increase so long as they continue living alone [18]. The majority of older adults face financial hardship during old age since most of them are not in a position to earn their living, which makes them feel lonelier and more isolated. Living alone results in a poor health status and also makes them prone to problems related to mental health [19,20]. The elderly living in isolation also encounter more problems of depression, despair, worthlessness, and having no life satisfaction than the older adults in other living modes [21,22]. Living alone, social isolation, and loneliness occur in approximately one-third or more of older adults, with 5% often or always feeling lonely [23,24]. Management of loneliness, isolation, and living alone requires medical and social interventions.

Living alone and social isolation among older adults in Thailand is the same as in many countries worldwide; it has risen and is expected to increase [25,26,27,28]. Currently, almost half of households have older people living in their households. However, the proportion of the older adults who live alone showed a remarkable increase at the same time. According to the report on the situation of the older adults in Thailand in 2020, out of a total of 21.9 million households, there are 1.3 million households (6%) with older adults living alone and another 1.4 million households (6.2%) have older adults living together without people of other ages living in the house. This means that more than 12% of older adults live alone [28]. The situation in Thailand has led to numerous physical, mental, economic, and housing problems. Furthermore, the study of the health status of older adults in Sweden confirmed that loneliness that results in a negative health status in the lives of older adults is associated with being alone. In other words, older adults who are alone for a long time will have poor health conditions with high loneliness [29]. Furthermore, the study of older adults living alone in Southeast Asia (e.g., Myanmar, Vietnam, and Thailand) provided interesting findings that demonstrated older adults living alone and without children are more vulnerable than older adults in other living schemes in terms of economics, mental health, and lack of attention [30].

This trend highlights the growing demand for home and community care and services, especially for the older adults who cannot help themselves, including supporting various infrastructure that will enable those still in good health to be able to live on their own. Therefore, this research aims to explore the evidence base for the determinants of the living characteristics of older adults living alone, with their health issues and daily activities, to understand the risks inherent in living with family or living with others. This will lead to a policy plan to deal with the problem of health and health inequities among older adults living alone, which is anticipated to increase in the future.

## 2. Literature Reviews

### 2.1. Ageing and Health

Globally, people who live longer are expected to live into their 60s and beyond. When people are in their 60s and beyond, they are called elders. The United Nations defines an older person as a person who is over 60 years of age. However, families and communities often use other socio-cultural referents to define age, including family status (grandparents), physical appearance, or age-related health conditions [31]. Currently, the number of people aged 60 years and above will outnumber children younger in 2030, and one in every six people worldwide is expected to be aged 60 years or over. The pace of population aging is much faster than in the past [32]. Notably, it has led to an aging society, affecting economic growth, work and retirement patterns, the way family’s function, the ability of governments and communities to provide adequate resources for older adults, and the prevalence of chronic disease and disability [33]. An aging society is one of the challenging issues in the world, stemming from medical advancement. The World Health Organization (WHO) developed a definition of healthy aging as the process of developing and maintaining the functional ability that enables well-being in older age [34,35,36]. At the biological level, aging results from the accumulation of a wide variety of molecular and cellular damage over time [32]. As time goes on, those who grow older usually come along with an increased risk of health problems related to physical and mental health. Aging presents significant challenges to public health and service needs, social interaction needs, and housing needs.

### 2.2. Living Alone and Isolation among Older Adults

Household living arrangements refers to whether or not a person lives with another person or persons—if so, whether or not he or she is related to that person or persons. The structure and composition of one’s household refers to all persons living together in a housing unit, including the number of household members and their relationships to each another [37,38]. Living arrangements in a household are a matter of household structure and can be divided into varieties [39,40]. If the household living patterns are divided according to the type of members who live together, they can be divided into six types: single households, households in which members are not related by kinship, one-generation households, two-generation households, three and more generations in a household, and aged and skipped-generation households [41]. The increasing situation of older adults has resulted in changes in the population structure in many countries. These demographic changes resulted in a difference in the population’s age structure from the past. These variations also influence the trend of population structure changes and the living arrangements of households, especially the living arrangements in households with or without older adults.

The household living arrangements of older persons can produce important implications for their health, economic status, physical and psychosocial health, life satisfaction, and well-being [42,43,44]. The living arrangements in a household with one person is a single household or living alone, and it has at least one household member with age of 60 years and over. It can be divided into several categories such as living alone, living together with older adults, living with children, living with working-age people, living with children and working-age people, living with spouse only, and living with others (co-residence) [45,46]. Living alone in a one-person household by older adults or living by themselves has risen and is expected to increase more [11,12,17,47]. Living alone at older ages is associated with increased mortality risk and this phenomenon (living alone) is related to being socially isolated and feeling lonelier [48,49,50].

Social isolation is considered to be an objective measure of limited social contact (number of personal contacts) between an individual and society [51]. Social isolation is often measured based on social network size, diversity, or frequency of social activity [52,53]. People suffering from social isolation are at an increasing risk of overt diabetes, coronary heart disease, dementia, and a rise in the all-cause mortality rate [54]. Older adults often suffer from health problems and disabilities; perhaps older adults living alone are more likely to experience emotional problems that are due to social isolation [55,56]. Challenges faced by older adults living alone may be correlated with various factors, including the death of a spouse, divorce, poorer physical or mental health, and personal choices that had several relevant factors, as summarized from the research reviews in Table 1 [57].

For older adults living alone, there is an issue that should be paid attention to, which is the issue of caring for the older adults to enable them to live independently with stability, safety, and confidence in their daily lives in the future.

## 3. Materials and Methods

### 3.1. Study Area

**Figure 1:**
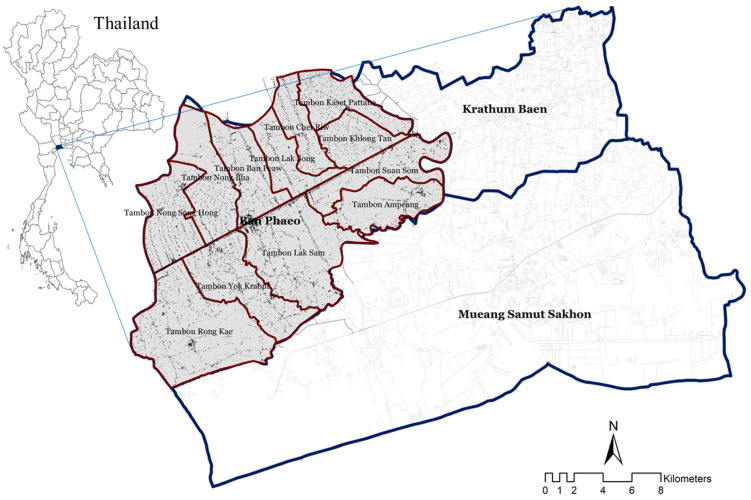
Study area: Ban Phaeo district, Samut Sakhon province, Thailand.

The study area that is focused on in this study is Ban Phaeo district, Samut Sakhon province, Thailand, as depicted in Figure 1. As for the characteristics of Ban Phaeo district, the terrain is a coastal plain, consisting of a district divided into two parts by the Damnoen Saduak canal and 12 sub-districts. The area topographies of its soil fertility and water resources interconnected with river networks spread throughout the area, in both the natural and canal areas. The canal was dug to bring fresh water for cultivation and irrigation. It also helps in drainage and transportation, thereby making the area suitable to cultivate various plants, as well as some businesses, with industrial and residential areas. The settlement of Ban Phaeo is still a low-density settlement with over 90% of the total area regarded as agricultural and natural areas. Exceptionally, Krathum Baen and Mueang Samut Sakhon districts have more concentration amongst the settlements. Settlements affect the livelihoods of older adults in terms of access to public health services and social interaction, especially the dispersed settlement areas. This is causing older adults to choose living at home rather than participating in social activities because of the relatively long distance and difficulty traveling [73,74]. These older adults at home are still considered as a group that can still participate in social activities, although sometimes requires the help of others [75,76]. Consequent upon the dispersed physical nature of the settlements and the distance from the activity areas, these older adults decide to participate less unless children, grandchildren, or caregivers are there to facilitate their movement [77]. This shows that when a group of older adults live alone with no children, they are less likely to participate in social activities.

### 3.2. Data Collection

**Figure 2:**
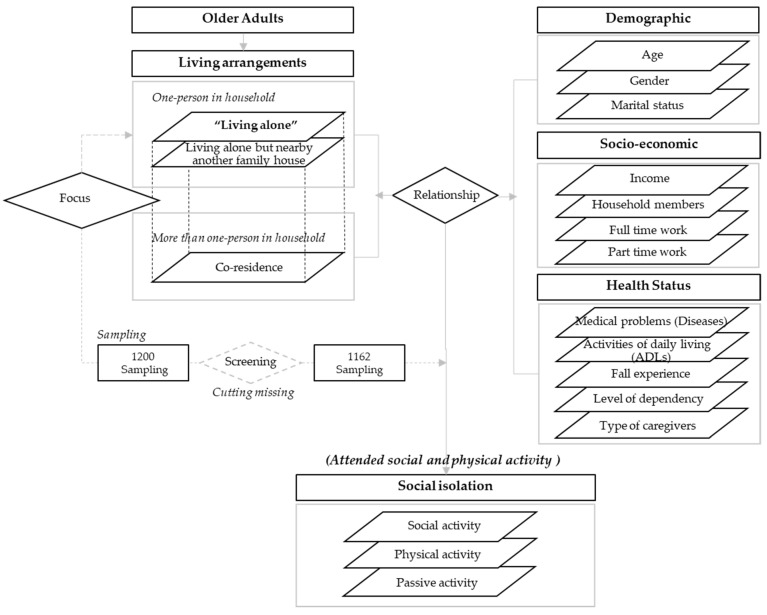
Framework of study.

The samples used in this article focused on older adults aged 60 and above and were restricted to respondents living in Ban Phaeo, Samutsakhon, Thailand. The sample was older adults with normal communication ability and excluded those who have psychological health problems. The target group is older adults who live alone and in co-residence (living together). The sample size of older adults was calculated from a total population of 97,005 persons living in the Ban Phaeo district, according to 2017 population data. The sample size was determined by using a sampling error of 0.05, with 400 samples as the minimum number of sample groups. Therefore, the data collection was designed to cover the sample groups with a variety of living arrangement, socio-demographic, health status, and social isolation types within the study area. Furthermore, based on the older adults and population aging statistics in the study area, it aimed to gather at least 1200 sets. After data cleaning and the screening of missing data, the remaining data used as input for further analysis process amounted to 1162 samples. Five issues were considered (as illustrated in Figure 2), which consisted of the following factors:

(1) Living arrangement: represents one factor of “type of living arrangements”;
(2) Demographic: presents three factors of “age, gender, marital status”;
(3) Socio-economic: consists of three factors of “income, number of household members, working status (full time and part time)”;
(4) Health status: covers five factors of “medical problems (number of chronic diseases), activities of daily living (ADLs), fall experience, level of dependency and type of caregivers”;
(5) Social isolation: consists of one factor of “attended social and physical activity (passive activity, physical activity and social activity)”.

This data collection was conducted by questionnaire survey and the questionnaire was approved by the Institutional Review Board (IRB) of Thammasat University (approval number 200/2017) for consideration in the International Conference on Harmonization—Good Clinical Practice (ICH-GCP). The sampling had written-consent approval from the target group before the commencement of the face-to-face surveys conducted at the homes of the older adults.

### 3.3. Data Analysis

There are a number of factors related to an older adult’s lonely living. However, the outcomes of the significant factors in particular research also differed because of the nature of the data of the imported samples as a result of the different contexts of each area. In this regard, the study of relevant factors represented in a specific area is important for the planning that will accommodate its context, as outcomes from one area may not be applicable in all areas because of differences in the context of the data, regardless of demographic characteristics, social and economic characteristics, etc. [78,79,80]. Therefore, this study seeks to understand the factors associated with older adults living alone, including whether the older adults’ being alone affects their participation in social activities. Meanwhile, participating in social and physical activities is a significant risk situation of social isolation for older adults living alone.

Statistical analyses were applied by using descriptive statistics and regression analysis through the application of SPSS statistics (version 28.0) Descriptive analyses were performed for all variables by employing correlations to assess the associations between living alone and other variables (demographic, socio-economic, health status, and social isolation) with descriptive statistics (e.g., mean, SD, and percentage). Furthermore, the binary logistic regression model was used to investigate the association between the characteristics of living alone and social isolation. For each variable outcome, three models were estimated. Model 1 regressed the combinations of the demographic of living alone on the outcome variables (social isolation) as the base model. Model 2 repeated the analyses, while including the adjusting of the socio-economic status of living alone. Finally, Model 3 repeated the analyses while incorporating the adjusting of the health status of living alone. All tests were evaluated at a 0.05 level of statistical significance. This study examined how baseline variables explained the association between living alone and social isolation characteristics by calculating the percentage of excess variables.

## 4. Results

This study examined how baseline variables explained the association between living alone and social isolation characteristics by calculating the percentage of excess variables. The detail of the analysis among associated factors are explained as follows (see Table 2 and Table 3):

### 4.1. Characteristics of Older Adults

(1) Demographic characteristic

The study included 1162 older adult people living alone, representing 7% of the total older adult population (from the total of the older adult population of 16,614 persons in 2018). The living arrangement in the household of the sample group was divided into three categories by the following percentiles: 4.5% represents those living alone (one person in the household) and 4% accounted for those living alone but near their family; the remaining 91.5% accounted for co-residence (lived with family), i.e., those who may live together with their spouses, or with siblings, grandchildren, or others. From Table 2, the mean age (X_1_) of the entire population of this study is 73 years (SD = 9.27). This showed that the average age range is relatively high, and more than 60.7% of the samples are over 70 years old. The overall sample group alone accounts for 8.5% of the total sample, with females almost 63% (X_2_). For marital status (X_3_), 54.7% of the participants were married, followed by single (36.3%). When the factors related to older adults living alone was considered, it was found that more than 63.6% were females living alone. The mean age of these older adults who lived alone is 71 years (SD = 8.06), with over 55.5% being single. In addition to the consideration of different living arrangements with other variables, the marital status variable was also found to be significant and related to the differences in living arrangements.

(2) Socio-economic characteristic

In consideration of socio-economic factors (see Table 3), average monthly income (X_4_) less than THB 2000 was found to be over 68%, followed by THB 2000–5000 (16.4%) and THB 5001–10,000 (9.4%). The majority of the average monthly income for both older adult funding and low-income funding are from government support. Furthermore, it was found that the majority of the older adults are unemployed, with not even part-time jobs, e.g., farmers and general employment. Based on the above data analysis, it can be inferred that there is an association between the characteristics of urban activities with housing density and economic activities. The older adult group is likely to earn more in areas of densely populated urban activities. Being near the economic source provides more opportunities and access to income sources. However, the sources of income for older adults come from several sources, not just from the occupations of the older adults alone, but also from their children. However, in some cases, low-income older adults live alone without relatives or siblings, resulting to lower opportunity to access the basic services provided by the community, district, or province. The characteristics of the residential status showed the average number of household members (X_5_) are about 3–5 persons per household by indicating over 90% of the households have more than one person residing in them. For working status, these can be considered as two types for older adults’ earnings that are full-time (X_6_) and part-time (X_7_). The finding is illustrated that only 4.2% have full-time work; on the other hand, about 12.2% have part-time jobs. These issues indicated that most of the older adults represent no working group. The majority of older adults present their economically insecurity by struggling to meet their daily expenses and they mostly depend on the availability of social protection schemes or government welfare. When the factors relating to older adults living alone were considered, it was found that the number of households is one of the key variables for analysis related to the significance of different living arrangements.

(3) Health status

Decreased physical performance over time because of aging is a limitation in the daily lives of older adults, especially in their physical and mental health. Therefore, health planning is important for providing good health care, especially among older adults. This can be confirmed by the data collection and analysis of the health status of the older adults in Ban Phaeo district, which was explored by five factors (see Table 3). There are chronic diseases (X_8_), activities of daily living (ADLs) (X_9_), fall experiences (X_10_), level of dependency (X_11_), and type of caregiver (X_12_). The ADLs are derived from 10 issues, which comprise: (1) transfer (mean 3.40, SD 0.90), (2) mobility (mean 3.35, SD 0.94), (3) toilet use (mean 3.42, SD 0.89), (4) grooming (mean 3.46, SD 0.87), (5) bladder (mean 3.46, SD 0.88), (6) bowels (mean 3.46, SD 0.88), (7) bathing (mean 3.43, SD 0.89), (8) feeding (mean 3.52, SD 0.82), (9) dressing (mean 3.47, SD 0.87), and (10) stairs (mean 3.34, SD 0.97). Since the difficulties with activities of daily living provide a significant impact on older adults’ quality of life, ensuring daily physical activities can help prevent new and worsening health issues, which is significantly positive for longevity [65,66,67]. According to the collected data, most of the older adults in Ban Phaeo district have about 1–2 underlying diseases (X_8_). Over 73% of these diseases amongst all the older adults are chronic diseases, and 64% were chronic diseases in older adults living alone. General diseases involved blood pressure, diabetes, heart disease, kidney disease, memory loss, arthritis, bone, and skin. Considering the nature of the diseases, older adults develop most symptoms within about 2–10 years, but with advancing age, the status of the older adult group at home begins to increase the number of chronic diseases, as well as lead to declining mobility and physical function. When falls were considered (X_10_), 9.3% were recorded to have fallen, which is considered to be a relatively low proportion. However, the risk of falling occurs at any time when there is a lack of care, and falls can lead to injury and death [74,75,76]. More so, on the level of dependency (X_11_), over 80% were a group of independencies, followed by partially dependency (13%) and total dependency (7%). The type of caregivers (X_12_) was discovered, and it was found that the majority rely on their children (mean 4.36, SD 1.05), grandchildren (mean 3.70, SD 1.25), caregivers (private) (mean 3.64, SD 1.13), caregivers (volunteers) (mean 3.56, SD 1.06), and neighbors (mean 3.32, SD 1.04). Regarding activities of daily living (ADLs) (X_9_), the mean value of ADLs of this study is a 3.41 score (SD = 0.89). It was found that most of the older adults were capable of doing 80% of their routine activities. It reveals a moderate to high level of daily life activities by themselves; however, there are some older adults who cannot look after themselves. This is under the vulnerable group that needs to rely on others and is considered for different living arrangements with other variables. This shows that the level of dependency and type of caregiver variables are related to the significance of different living arrangements.

(4) Social isolation

When considering participation in activities and the ability to live in daily life among three ranges of age groups, namely 60–69 years, 70–79 years, and more than 80 years (see Table 4), it was found that the older adults who are in the early age group present better abilities to live their daily lives than those in the middle and older ages.

In terms of participating in various activities within the community, it was found that the older adults in the early age group were able to carry out their daily lives better than those in the middle and older age groups. Most of the older adults chose to perform passive and social activities more than other physical activities. The data suggests that as older adults age, their ability to maintain a daily routine declines with some difficulties and their ability to participate in activities decreases. This situation may be attributed to the decline in physical fitness with age. Particularly, older adults who lived alone present a higher risk of social isolation than those who live with others. When different living arrangements (living alone, living alone but near family, and living with others (co-residence)) considered their relationship with participation in social activities, the result showed that no variable is related to the significance of different unique living patterns (see Table 3). However, when social isolation among older adults living alone was considered (*X_13_*), the result demonstrated that only 8.9% attended social activities, 57.2% were passive activities and 33.9% attended the physical activities. The data shows that the older adults in this study had a relatively low level of participation in social activities, whether alone or with family members. Most of the daily activities of the older adults include talking to their neighbors and going to regular exercises, which they rarely participate in with others. Therefore, differences in living patterns were not statistically significant; as a result, the focus should be directed to further consideration of the influencing factors on living alone and the social isolation among the sample of the older adults who are alone and are participating in social activities. The details of the analysis are shown in the next section.

### 4.2. Relationship between Older Adults Living Alone with Other Factors

The results of the analysis of the relationship between older adults who live alone with other influencing factors showed that marital status, number of household members, and care by a child as caregiver was significant. This indicated that these factors are accordingly important to different forms of older adults, whether the person is single, unmarried, or divorced, and it can result in a single household (see Table 5).

However, the factors that resulted in a single household emanated from many supportive factors. In addition to household members, the numbers were significant because of the number of household members living alone that reflects the number of living arrangements in households. The cases were varied by having only one person in the household or co-residence (living with a family) and subsequently having more than one person, resulting in the number of household members being significant. Finally, the type of caregiver was significant when the older adults who live alone tend to have lower ability to rely on themselves because of physical deterioration. On the other hand, the older adults who live with their families, particularly children, tend to rely more on their family members, which allows them to feel less isolated and more positive about the care. In addition, as the type of caregivers is statistically significant with the older adults’ social isolation, it can be seen from the data that the dependency on their families plays an important role in supporting the older adults’ livelihoods. Considering the detail of the association between older adults living alone and social isolation, (i.e., attended social and physical activities), the further results of analysis are illustrated in Table 6.

For each outcome variable, three models were estimated. Model 1 presents the combinations of the demography of living alone on the outcome variables (social isolation) as the base model. With the analysis of the adjusted-for-demography variables in Model 1, it was found that age variable was significant (Exp(B) (X_1_) = 0.880; 95% CI= 0.828, 0.937), while gender and marital status were insignificant. Model 2 repeated the analyses by including adjusting the socio-economic variables. It was also verified that age was statistically significant (Exp(B) (X_1_) = 0.881; 95% CI = 0.825,0.941), and other factors were insignificant. Model 3 also repeated the analyses by adjusting the health status. It was confirmed that older people (Exp(B) (X_1_) = 0.905; 95% CI = 0.837,0.979) have problems with their activities of daily living (ADLs) (Exp(B) (X_9_) = 6.329; 95% CI = 1.308, 30.636). Furthermore, support from their children (as caregivers) benefits both the physical and mental health of older parents for reducing social isolation and loneliness (Exp(B) (X_12a_) = 0.314; 95% CI = 0.105, 0.946). This is because these people hardly go out to perform activities outside their home and rarely attend social and physical activities. With advancing age, an increasing number of major life adjustments results in them becoming dependent on others in their activities of daily living (ADLs), which consequently leads to a negative impact on life satisfaction.

## 5. Discussion

From the analysis, it was discovered that older adults have problems with their ability to carry out daily life; they may not participate in most activities. On the other hand, if they are in good health and not too old, they are more likely to participate in activities from the analysis of different living arrangements against other variables. It showed that the variables of marital status, household members numbers, level of dependency, and type of caregiver (child, grandchild, and neighbor) are related to the significance of different living arrangements. Many studies have shown that living alone is a risk factor for social isolation [81,82,83]. However, several studies argued that, in some cases, older adults who live alone might not feel lonely and isolated from society. Some of the characteristics of older adults who live alone were also found to be significant in terms of similar variables (e.g., marital status, number of household members, and level of dependency). Several studies demonstrated that older adults’ likelihood of living alone plays a key role as an influencing factor. Katz, Kabeto, and Langa (2000) revealed that older adults’ likelihood of living alone is influenced by several factors, including their demographic characteristics, current health status, and social, cultural, and environmental factors [58]. Individuals’ gender, marital status, race, and the number of children directly influence whether they would live alone as a senior adult. For instance, older women are more likely to live alone than their male counterparts.

Living alone in older adults is a notable issue. Several examples of research concerning aging in Singapore and Japan indicated that lonely older adults spent less of their remaining life in good health or being active compared to others [84]. Moreover, older adults tend to have difficulties in managing their daily activities, which restrict self-determination on their abilities and opportunities to perform ADLs independent of others. Inadequate social support places an older adult at the greatest risk of dying, particularly for those living alone. For instance, Berkman and Syme (1979) in the Alameda County longitudinal study found that the most isolated adults with the least amount of social contact were more than two times likely to die (men were 2.3 and women were 2.8) during the nine-year follow-up [85]. Finally, one of the most influential factors that determines the quality of life among aging adults is isolation and living alone. Lack of fulfillment of personal relationships affects not just mental and emotional health, but also their quality of life and well-being [76,78].

## 6. Conclusions

An increasing number of older adults poses the likelihood or risk of being socially isolated or lonely. Many older adults who live alone may be lonely, although maybe not all of them. However, this phenomenon of a rapidly increasing number of older adults should be understood and planned for to prevent all of the associated risks. This study highlighted the issue of several studies about older adults living alone with social isolation, which was found to be a major influencing factor relative to different living arrangements of older adults amongst other factors (e.g., marital status, number of household members, level of dependency, and type of caregiver). A correlation was found between the characteristics of living alone and social isolation along three variables that relates to the characteristics of living alone and social isolation. These three variables are age, activities of daily living (ADLs), and type of caregiver. When only a group of older adults living alone with social isolation is considered, it was noticed that older adults living alone with advanced age present the most susceptibility to the damage of social isolation. Furthermore, the results revealed that older adults have problems with the activities of daily living (ADLs), resulting in a more sedentary lifestyle and health problems. These older groups often do not go out to do activities outside their homes and attend fewer social and physical activities. This situation could lead to the risk of social isolation, which causes a greater risk of physical and mental health problems. This effect could impact on the well-being of older adults who will live alone in the near future. In conclusion, it was found that different living arrangements in the household and social isolation are influenced by different associated factors, particularly type of caregiver, with the tendency that older adults receiving care from their children will benefit by maintaining a better health status. Importantly, this study provides better understanding of how needs emerge to improve the health and well-being in the cases of loneliness among the older adults in rural areas of Thailand. The present of barriers for healthy aging in terms of physical environment and social environment demonstrate an essential insight that can be applied in other contexts of rural settings as a guide for the identification of older adults’ needs. To foster healthy aging, the levels of dependency and its associated factors in living alone and social isolation among older adults in rural areas are required to shape context-sensitive measurements and policy interventions to counter social isolation in later life.

In addition to tackling living alone and social isolation, found in some of the literature about understanding this association, this study could provide perspectives of older adults with various demographic and socio-economic under different health statuses. To provide inclusive understanding for promoting health and preventing disabilities, the involvement among older adults, caregivers, and household characteristics must be identified, specifically, with the importance of participation in social activity. Both personal and environmental facilitators can promote the needs of older adults in social participation according to older adults’ specific perspectives (needed assistance, physical accessibility, adapted activities, and local transportation). However, this study has limitations that are due to the lack of consideration of the older adults’ mental health and living environments. Mainly, loneliness and isolation are related to older adults’ mental and environmental conditions. Such different tangible attributes could help to explain significant issues for older adults’ needs and to address these critical challenges to promote social participation. However, social participation needs may vary with the context of the community since rural communities are not all homogeneous and the diversities on the typology of the sites requires a multisite approach. The proximity to the geography of aging presents as a means of differentiating between rural areas, which could help to demonstrate distinct combinations of demographic, socioeconomic, and policy challenges across rural areas. Therefore, to make future research more comprehensive, such considerations should be taken to cover both physical and psychological factors among older adults to recommend sustainable coping strategies in maintaining their levels of independence. Interventions on determinants of health should be used to set a policy agenda that focuses on fostering age-friendly environments and facilitates physical activity for all age groups, especially among older adults. Implementation of such initiatives may finally reduce the isolation and vulnerability of older adults living in rural areas, while increasing their independent life expectancy toward a better quality of life. Finally, it is believed that the outcome of this research would provide valuable support for health and social service interventions targeting isolated seniors or seniors at risk of being isolated to improve their health outcomes, quality of life, and well-being.

## Figures and Tables

**Table 1:** Factors correlated to living alone in older adults.

Items	Detail	References
Demographic	Marital status	[16,57,58,59,60,61,62]
	Gender	[58,59,63]
	Race	[58,59]
Health status	Physical or mental health status	[57,64,65]
	Activities of daily living (ADLs)	[65,66,67]
Economic	Income	[16,67,68]
Social	Personal choice	[57]
	Prefer to live independently	[16,17]
	Number of living children	[58,59]
	Older adults who never had a child	[61]
	Person’s level of education	[69,70,71,72]

**Table 2:** The demographic and socio-economic characteristics of older adults.

Variables		Living Alone	Living Alone but Near Family	Living with Others (Co-Residence)	Total	*p*-Value
**Demographic**						
Age (*X*_1_) (years, mean ± SD)		71.35 (±8.06)	74.28 (±9.39)	73.12 (±9.32)	73.09 (±9.27)	0.379
Gender (*X*_2_) (n/%)	Male	23 (2.0%)	13 (1.1%)	394 (33.9%)	430 (37.0%)	0.231
	Female	29 (2.5%)	34 (2.9%)	669 (57.6%)	732 (63.0%)	
Marital status (*X*_3_) (n/%)	Married	9 (0.8%)	17 (1.5%)	610 (52.5%)	636 (54.7%)	0.000 **
	Divorce	11 (0.9%)	7 (0.6%)	86 (7.4%)	104 (9.0%)	
	Single	32 (2.8%)	23 (2.0%)	367 (31.6%)	422 (36.3%)	
**Socio-economic**						
Average Income (*X*_4_) (THB) (n/%)	Less than 2000 (USD 61.9)	37 (3.2%)	33 (2.8%)	730 (62.8%)	800 (68.8%)	0.621
	2000–5000 (USD 61.9–154.8)	7 (0.6%)	7 (0.6%)	176 (15.1%)	190 (16.4%)	
	5001–10,000 (USD 154.8–309.5)	8 (0.7%)	5 (0.4%)	96 (8.3%)	109 (9.4%)	
	10,001–15,000 (USD 309.5–464.3)	0 (0.0%)	2 (0.2%)	47 (4.0%)	49 (4.2%)	
	More than 15,000 (>USD 464.3)	0 (0.0%)	0 (0.0%)	14 (1.2%)	14 (01.2%)	
Number of household members (*X*_5_) (n/%)	1 person	52 (4.5%)	35 (3.0%)	19 (1.6%)	106 (9.2%)	0.000 **
	More than 1 person	0 (0.0%)	11 (1.0%)	1039 (89.9%)	1050 (90.8%)	
Full time working status (*X*_6_) (n/%)	Yes	3 (0.3%)	0 (0.0%)	46 (4.0%)	49 (4.2%)	0.299
	No	49 (4.2%)	47 (4.0%)	1017 (87.5%)	1113 (95.8%)	
Part time working status (*X*_7_) (n/%)	Yes	7 (0.6%)	3 (0.3%)	132 (11.4%)	142 (12.2%)	0.448
	No	45 (3.9%)	44 (3.8%)	931 (80.1%)	1020 (87.8%)	

Note: 1162 sets; ** = tests were evaluated at a 0.01 level of statistical significance; 1 United States dollar (USD) was 32.3104 baht (THB) in 2018, source from Bank of Thailand (2022).

**Table 3:** Health status and social isolation of older adults.

Variables		Living Alone	Living Alone but Near Family	Living with Others (Co-Residence)	Total	*p*-Value
**Health Status**						
Number of chronic diseases (*X*_8_) (n/%)	No chronic disease	15 (0.6%)	20 (0.9%)	279 (12.0%)	314 (27.0%)	0.204
	1 chronic disease	19 (0.8%)	12 (0.5%)	392 (16.9%)	423 (36.4%)	
	2 chronic diseases	12 (0.5%)	8 (0.3%)	269 (11.6%)	289 (24.9%)	
	More 2 chronic diseases	6 (0.3%)	7 (0.3%)	123 (5.3%)	136 (11.7%)	
Activities of daily living (ADLs) (*X*_9_) (mean ± SD)	Transfer	3.40 (±0.82)	3.45 (±0.86)	3.40 (±0.91)	3.40 (±0.90)	0.315
	Mobility	3.37 (±0.84)	3.43 (±0.88)	3.35 (±0.94)	3.35 (±0.94)	
	Toilets use	3.48 (±0.70)	3.51 (±0.75)	3.42 (±0.90)	3.42 (±0.89)	
	Grooming	3.58 (±0.61)	3.53 (±0.75)	3.46 (±0.88)	3.46 (±0.87)	
	Bladder control	3.56 (±0.61)	3.53 (±0.75)	3.45 (±0.89)	3.46 (±0.88)	
	Bowels control	3.56 (±0.61)	3.53 (±0.75)	3.45 (±0.89)	3.46 (±0.88)	
	Bathing	3.54 (±0.64)	3.51 (±0.75)	3.43 (±0.90)	3.43 (±0.89)	
	Feeding	3.58 (±0.61)	3.57 (±065)	3.51 (±0.84)	3.52 (±0.82)	
	Dressing	3.58 (±0.61)	3.53 (±0.75)	3.46 (±0.88)	3.47 (±0.87)	
	Stair climbing	3.38 (±0.82)	3.49 (±0.78)	3.33 (±0.99)	3.34 (±0.97)	
	ADLs (mean ± SD)	3.50 (±0.63)	3.51 (±0.74)	3.41 (±0.91)	3.41 (±0.89)	
Fall experience (*X*_10_) (n/%)	No	47 (4.0%)	39 (3.4%)	968 (83.3%)	1054 (90.7%)	0.174
	Yes	5 (0.4%)	8 (0.7%)	95 (8.2%)	108 (9.3%)	
Level of dependency (*X*_11_) (n/%)	Independency	47 (4.0%)	34 (2.9%)	851 (73.2%)	932 (80.2%)	0.014 *
	Partially dependency	5 (0.4%)	12 (1.0%)	136 (11.7%)	153 (13.2%)	
	Total dependency	0 (0.0%)	1 (0.1%)	76 (6.5%)	77 (6.6%)	
Type of caregivers (*X*_12_) (mean ± SD)	Child	3.25 (±1.40)	3.83 (±1.34)	4.44 (±0.98)	4.36 (±1.05)	0.000 **
	Grandchild	2.94 (±1.34)	3.43 (±1.50)	3.75 (±1.22)	3.70 (±1.25)	0.000 **
	Neighbor	3.00 (±1.21)	3.30 (±1.20)	3.33 (±1.03)	3.32 (±1.04)	0.002 **
	Doctor or public health officer	3.04 (±1.19)	3.13 (±1.24)	3.24 (±1.06)	3.22 (±1.08)	0.094
	Volunteers	3.44 (±1.24)	3.51 (±1.20)	3.57 (±1.04)	3.56 (±1.06)	0.149
	Caregiver (private)	3.29 (±1.27)	3.43 (±1.25)	3.67 (±1.12)	3.64 (±1.13)	0.081
	Others	2.94 (±1.27)	3.19 (±1.28)	3.31 (±1.23)	3.29 (±1.23)	0.042 *
**Social isolation**						
Social isolation (*X*_13_) (n/%)	Passive activity	21 (4.2%)	27 (5.4%)	450 (90.4%)	498 (57.2%)	0.746
	Physical activity	14 (4.7%)	14 (4.6%)	267 (90.5%)	295 (33.9%)	0.116
	Social activity	2 (2.3%)	7 (8.1%)	77 (89.5%)	77 (8.9%)	0.089

Note: 1162 sets; * = tests were evaluated at a 0.05 level of statistical significance; ** = tests were evaluated at a 0.01 level of statistical significance.

**Table 4:** Participation in activities among older adults.

Variables		60–69 Years	70–79 Years	More than 79 Years	Total	*p*-Value
ADLs (mean ± SD)		3.81 (±0.51)	3.41 (±0.82)	2.86 (±0.98)	3.36 (±0.48)	0.000 **
Participation in activities	Passive activity	301 (12.5%)	221 (9.2%)	94 (3.9%)	616 (25.6%)	0.000 **
	Physical activity	147 (6.1%)	98 (4.1%)	50 (2.1%)	295 (12.3%)	0.000 **
	Social activity	268 (11.1%)	158 (6.6%)	73 (3.0%)	499 (20.7%)	0.000 **

Note: 1162 sets; ** = tests were evaluated at a 0.01 level of statistical significance.

**Table 5:** Correlation of living alone with characteristics of older adults (demographic, socio-economic, health status, and social isolation).

Variables	Living Alone		Living Alone but Near Family		Living with Others (Co-Residence)	
	Correlation	*p*-Value	Correlation	*p*-Value	Correlation	*p*-Value
**Demographic**						
Age (*X*_1_)	−0.041	0.166	0.026	0.369	0.012	0.695
Gender (*X*_2_)	−0.032	0.270	0.040	0.176	−0.004	0.890
Marital status (*X*_3_)	0.145	0.000 **	0.068	0.020 *	−0.156	0.000 **
**Socio-economic**						
Average income (THB) (*X*_4_)	−0.021	0.482	−0.009	0.752	0.022	0.457
Number of household members (*X*_5_)	−0.304	0.000 **	−0.212	0.000 **	0.375	0.000 **
Full time working status (*X*_6_)	0.017	0.569	−0.043	0.142	0.018	0.539
Part time working status (*X*_7_)	0.008	0.780	−0.037	0.213	0.020	0.501
**Health Status**						
Number of chronic diseases (*X*_8_)	−0.012	0.680	−0.038	0.196	0.036	0.223
Activities of daily living (ADLs)(*X*_9_)	0.022	0.462	0.022	0.453	−0.032	0.283
Fall experience (*X*_10_)	0.003	0.917	0.040	0.169	−0.031	0.295
Level of dependency (*X*_11_)	−0.064	0.030 *	0.012	0.681	0.039	0.189
Type of caregiver: Child (*X*_12*a*_)	−0.229	0.000 **	−0.103	0.000 **	0.242	0.000 **
Type of caregiver: Grandchild (*X*_12*b*_)	−0.131	0.000 **	−0.045	0.125	0.129	0.000 **
Type of caregiver: Neighbor (*X*_12*c*_)	−0.066	0.024 **	−0.004	0.895	0.052	0.078
Type of caregiver: Doctor or public health officer (*X*_12*d*_)	−0.037	0.203	−0.018	0.529	0.041	0.165
Type of caregiver: Volunteer (*X*_12*e*_)	−0.025	0.394	−0.010	0.722	0.026	0.378
Type of caregiver: Caregiver (private) (*X*_12*f*_)	−0.067	0.022 **	−0.039	0.187	0.077	0.009 **
**Social isolation**						
Passive activity (*X*_13*a*_)	−0.022	0.462	−0.017	0.564	0.028	0.341
Physical activity (*X*_13*b*_)	0.008	0.795	0.0021	0.480	−0.020	0.489
Social activity (*X*_13*c*_)	−0.029	0.317	0.059	0.045*	−0.020	0.502

Note: * = tests were evaluated at a 0.05 level of statistical significance; ** = tests were evaluated at a 0.01 level of statistical significance.

**Table 6:** Association between living alone and social isolation.

Variables	Model 1			Model 2			Model 3		
	S.E.	*p*	Exp (B) (95% C.I)	S.E.	*p*	Exp (B) (95% C.I)	S.E.	*p*	Exp (B) (95% C.I)
**Demographic**									
Age (*X*_1_)	0.032	0.000 **	0.880 (0.828, 0.937)	0.034	0.000 **	0.881 (0.825, 0.941)	0.040	0.013 *	0.905 (0.837, 0.979)
Gender (*X*_2_)	0.515	0.087	2.410 (0.879, 6.608)	0.543	0.063	2.742 (0.947, 7.943)	0.678	0.070	3.415 (0.904, 12.896)
Marital status (*X*_3_)	0.282	0.133	1.527 (0.879, 2.652)	0.310	0.198	1.490 (0.812, 2734)	0.377	0.096	1.873 (0.895, 3.921)
**Socio-economic**									
Average income (THB) (*X*_4_)				0.403	0.495	1.316 (0.597, 2.901)	0.499	0.976	0.985 (0.370, 2.621)
Number of household members (*X*_5_)				0.193	0.736	1.067 (0.731, 1.560)	0.306	0.663	1.143 (0.627, 2.080)
Full time working status (*X*_6_)				1.423	0.072	0.77 (0.005, 1.257)	2.017	0.261	0.104 (0.002, 5.400)
Part time working status (*X*_7_)				1.081	0.572	1.844 (0.221, 15.352)	1.573	0.210	7.176 (0.329, 15.752)
**Health Status**									
Number of chronic diseases (*X*_8_*)*							0.327	0.222	0.670 (0.353, 1.273)
Activities of daily living (ADLs) (*X*_9_)							0.805	0.022 *	6.329 (1.308, 30.636)
Fall experience (*X*_10_)							1.015	0.303	2.846 (0.390, 20.786)
Level of dependency (*X*_11_)							1.050	0.500	2.032 (0.259, 15.927)
Type of caregiver: Child (*X*_12*a*_)							0.562	0.040 *	0.314 (0.105, 0.946)
Type of caregiver: Grandchild (*X*_12*b*_)							0.312	0.611	0.853 (0.462, 1.574)
Type of caregiver: Neighbor (*X*_12*c*_)							0.479	0.636	1.254 (0.491, 3.205)
Type of caregiver: Doctor or public health officer (*X*_12*d*_)							0.320	0.464	0.791 (0.422, 1.482)
Type of caregiver: Volunteer (*X*_12*e*_)							0.471	0.119	2.084 (0.828, 5.246)
Type of caregiver: Caregiver (private) (*X*_12*f*_)							0.619	0.258	2.013 (0.599, 6.769)

Note: ADLs = activities of daily living; * = tests were evaluated at a 0.05 level of statistical significance; ** = tests were evaluated at a 0.01 level of statistical significance.
